# Nitric Oxide Mediates Stretch-Induced Ca2+ Release via Activation of Phosphatidylinositol 3-Kinase-Akt Pathway in Smooth Muscle

**DOI:** 10.1371/journal.pone.0002526

**Published:** 2008-06-25

**Authors:** Bin Wei, Zheng Chen, Xu Zhang, Morris Feldman, Xian-zhi Dong, Robert Doran, Bao-Lu Zhao, Wen-xuan Yin, Michael I. Kotlikoff, Guangju Ji

**Affiliations:** 1 Institute of Biophysics, Chinese Academy of Sciences, Beijing, China; 2 Department of Biomedical Sciences, College of Veterinary Medicine, Cornell University, Ithaca, New York, United States of America; University of Arkansas, United States of America

## Abstract

**Background:**

Hollow smooth muscle organs such as the bladder undergo significant changes in wall tension associated with filling and distension, with attendant changes in muscle tone. Our previous study indicated that stretch induces Ca^2+^ release occurs in the form of Ca^2+^ sparks and Ca^2+^ waves in urinary bladder myocytes. While, the mechanism underlying stretch-induced Ca2+ release in smooth muscle is unknown.

**Methodology/Principal Findings:**

We examined the transduction mechanism linking cell stretch to Ca^2+^ release. The probability and frequency of Ca^2+^ sparks induced by stretch were closely related to the extent of cell extension and the time that the stretch was maintained. Experiments in tissues and single myocytes indicated that mechanical stretch significantly increases the production of nitric oxide (NO) and the amplitude and duration of muscle contraction. Stretch- induced Ca^2+^ sparks and contractility increases were abrogated by the NO inhibitor L-NAME and were also absent in eNOS knockout mice. Furthermore, exposure of eNOS null mice to exogenously generated NO induced Ca^2+^ sparks. The soluble guanylyl cyclase inhibitor ODQ did not inhibit SICR, but this process was effectively blocked by the PI3 kinase inhibitors LY494002 and wortmannin; the phosphorylation of Akt and eNOS were up-regulated by 204±28.6% and 258±36.8% by stretch, respectively. Moreover, stretch significantly increased the eNOS protein expression level.

**Conclusions/Significance:**

Taking together, these results suggest that stretch-induced Ca2+ release is NO dependent, resulting from the activation of PI3K/Akt pathway in smooth muscle.

## Introduction

It has long been known that increases in passive tension evoke contraction of smooth muscle in arteries [1], and evidence suggests that the underlying mechanism involves graded increases in Ca^2+^ within vascular smooth muscle cells as arteries are pressurized [2]. Similarly, hollow smooth muscle organs such as the bladder, gallbladder, and gastrointestine undergo significant changes in wall tension associated with filling and distension, with attendant changes in muscle tone. Previously we demonstrated that increases in cell length trigger the gating of ryanodine receptor (RYR) Ca^2+^ release channels, resulting in a release of Ca^2+^ from the SR in the form of Ca^2+^ sparks or propagated Ca^2+^ waves. This stretch -induced Ca2+ release (SICR) process does not require an influx of extracellular Ca^2+^ ions, activation of ionic currents, or even a rise in [Ca^2+^]_i_ [3], despite the fact that stretch of smooth muscle cells may activate non-selective cation channels [4]–[7]. Here we examine the mechanism leading to Ca2+ release following stretch of urinary bladder smooth muscle cells.

NO is a cellular second messenger that mediates numerous biological functions such as vasodilation, muscle contractility [8], [9], anti-apoptosis [10], heart rate, and heart development [11], [12]. Three isoforms of nitric oxide synthase (NOS) catalyze NO formation, with eNOS accounting for most production in vascular and non-vascular smooth muscle [13]. Evidence suggests that stretch is associated with increased tissue NO formation in cardiac muscle [14]–[16], which may act to alter force production through effects on Ca^2+^ release by SR ryanodine receptors (RYR) [16], [17]–[20]. NOS activation by stretch of cardiac muscle is mediated by activation of the PI(3)K−Akt−endothelial NOS axis, and contributes to myocardial contractile activation during heart stretch. Recently we demonstrated that cell stretch induces Ca^2+^ release in the form of Ca^2+^ sparks in smooth muscle cells isolated from the urinary bladder [3]. The upstream transduction mechanism linking cell elongation to Ca^2+^ release in smooth muscle is not known, however. In the present study we investigated the signaling pathways mediating stretch-induced Ca2+ release in smooth muscle. We report that smooth muscle stretch enhances NO production and that this increase correlates with augmented RYR -mediated Ca2+ sparks. The production of NO is both necessary and sufficient to trigger Ca2+ sparks, as stretch –induced Ca2+ sparks were not activated under conditions of NOS inhibition or in eNOS knockout smooth muscle, and exogenous NO restored stretch –induced Ca^2+^ release in eNOS knockout cells, indicating that, NO mediates stretch-induced Ca2+ release through activation of PI(3)K-Akt−endothelial NOS axis in smooth muscle.

## Materials and Methods

### Single Cell and Tissue Strip Preparation

Mice including eNOS knockout mice that were obtained from the Jackson Laboratories (Bar Harbor, ME) were anesthetized and euthanized in accordance with an approved laboratory animal protocol of Cornell University and Chinese Academy of Sciences. Single cells were prepared as described previously [3]. Briefly, bladder myocytes were isolated by cutting the bladder into small pieces, which were incubated for 20 min in 1 mg/ml papain, 1 mg/ml dithioerythritol, and 1 mg/ml bovine serum albumin Ca^2+^-free solution. The fragments were then transferred into 1 mg/ml collagenase type II (Worthington Biochemical), and 100 µM Ca^2+^ solution, supplemented with 1 mg/ml bovine serum albumin. The tissue was incubated for 10 min, triturated with a wide-bore Pasteur pipette, and passed through 125-µm nylon mesh. Cells were concentrated by low speed centrifugation, washed with fresh medium, resuspended, and stored at 4°C.

Tissue segments were prepared by removing the fibrosal and mucosal layers from mouse bladders in ice-cold Ca^2+^ free solution, and cutting segments of the remaining muscle layer in 2–3 mm long strips, 100–200 µm in diameter, using a fine dissecting scissors. Tissue strips used for imaging were gently digested in 0.5 mg/ml collagenase type II with 1 mg/ml bovine serum albumin for 5 min at 32°C.

### Measurement of Ca^2+^ Fluorescence

**Figure 1:**
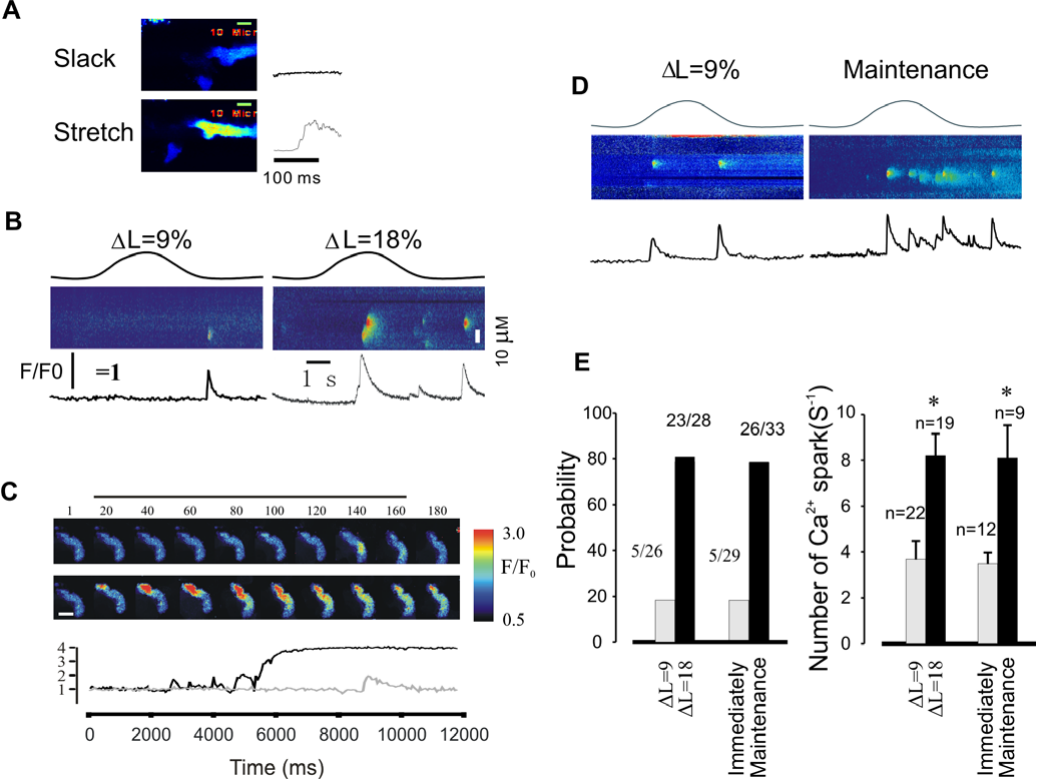
SICR is related to stretch length and maintaining time in single mouse bladder myocytes. A, elongation of whole cell images (left panel; scale bar 10 µM) demonstrates proportionate changes in length with cell stretch (upper at slack length without Ca2+ release and lower during stretch with Ca2+ release, respectively); the right panels are profiles from slack (upper) and stretch (lower). B, Linescan images obtained from different stretch length of myocytes show the alteration of Ca2+ spark property with the elongation of cells. C, Sample of x-y images recorded from different extent of stretch (ΔL = 9%, upper; ΔL = 18%, lower, respectively); the relative fluorescence intensity from stretch length ΔL = 9% (gray) and ΔL = 18% (black) shown under the images, respectively. The numbers above each image indicate the relative timing of acquisition, beginning just before the stretch to 9% or 18%. D,linescan images obtained from different time point after stretch: the left was obtained immediately after stretch, the right was recorded after stretch and maintained for 5 min. Note that at the time point after stretch and maintained for 5 min the Ca2+ spark frequency was increased significantly. E, summary data show the probability of cells with Ca2+ release follow different extend of stretch and time point (left) and the frequency of Ca2+ sparks (right). Note that the significant difference of the probability and the frequency of Ca2+ spark at different stretch length and different time point after stretch. Ns = number of experiments, * = P value less 0.05.

Single myocytes were incubated with 10 µM Fluo-4AM (Molecular Probes) for 10 min at room temperature in a recording chamber mounted on an inverted microscope (IX81, Olympus) and perfused with physiological salt solution for 40 min at room temperature. The extracellular solution was (mM): 140 NaCl, 5.4 KCl, 1.8 CaCl_2_, 1.2 MgCl_2_, 10 HEPES, and 10 glucose (pH 7.4, adjusted with NaOH). For the Ca^2+^-free extracellular solution, CaCl_2_ was omitted from the above solution or 3 mM EGTA and 1 mM CaCl_2_ was used to clamp free [Ca^2+^] at ∼100 nM, as indicated. Solutions were changed using a gravity perfusion system providing complete solution exchange within 30 s. Cell stretch was accomplished using two patch pipettes attached to separate manipulators (Fig. 1A). Pipettes were sealed to cells by suction to form a high resistance seal and cells were stretched by moving the pipettes along the longitudinal cell axis, stretching from slack length (L_1_) to a new length (L_2_) is reported as the percent increase in cell length, or (L_2_−L_1_)/L_1_. For experiments in tissue segments, the preparations were incubated with Fluo-4AM (10 µM) and 0.05% pluronic acid for 1 h. The strip was initially stretched by a few percent over slack length to approximate resting length. All experiments were conducted at room temperature. For CICR experiments the classical whole-cell voltage clamp technique was used. The intracellular solution was (mM): 130 CsCl, 1 MgCl_2_, 10 Hepes, 0.075 EGTA, 1 Mg-ATP (pH = 7.2, adjusted with CsOH).

Fluo-4 fluorescence was recorded using a laser scanning confocal head (FV1000; Olympus) attached to an inverted microscope (IX81; Olympus) with a plan-apo 60× water immersion objective lens. Cells were excited with 488 nm light from a krypton/argon laser and linescan or x-y images recorded using Fluoview software (Olympus). Linescans were obtained at an interval of 1.33 or 0.833 ms per line; x-y images (128 pixels×30 or 40 lines) were recorded at an average frame rate of 37.5-, 44-, or 57.3-ms intervals. Images were processed and analyzed using MATLAB 7.1 software (MathWorks). For linescan images, F_0_ was obtained by averaging the fluorescence for each pixel (x dimension) for a period preceding activation of a Ca^2+^ spark, and the fluorescence of all pixels (F) was divided by F_0_.

### Detection of NO by Electron Spin Resonance (ESR) in Tissues

Mouse bladders were removed and dissected as described above, and the muscle segments incubated in 30 mM diethyldithiocarbamate (DETC), 3 mM ferrous sulfate, and 15 mM sodium dithionite for 25 min at room temperature. To test stretch dependence of NO formation, tissue segments were elongated by about 30% of initial length and the stretched and control segments homogenized. The adduct (DETC)_2_-Fe2+-NO was extracted by ethyl acetate, and then measured on an ESR spectrometer at room temperature. The process was carried out in a dark environment to avoid light -induced NO dissociation from the adduct.

### Real Time Detection of NO

Cells and tissues were incubated with the NO-sensitive indicator 4, 5-diaminofluorescein (DAF-2). Cells were loaded with DAF-2 by incubation with 5 µM for 30 min, and tissue segments with the same concentration for 60 min, at room temperature. NO fluorescence was detected using a laser scanning confocal head (FV1000; Olympus) attached to an inverted microscope (excitation at 488 nm, emission at 515–565 nm).

### Contractility Studies

Contractility of isolated bladder smooth muscle segments was measured using a MyoMED myograph system (MED Associates, Inc., Georgia, VT, USA). Strips were mounted in a tissue bath containing aerated PSS (5 ml volume, 95% O_2_ and 5% CO_2_, 37°C) and stretched to 0.2 g (as control) and 0.5 g (as stretch) of tension, and stabilized for 45 min before beginning experimental protocols. Contraction amplitude and frequency were analyzed.

### Phosporylation of Akt and eNOS in Smooth Myocytes

For the measurement of phosphorylation and expression level of Akt and eNOS, bladder smooth muscle tissues were subjected to 30∼50% stretch in oxygenated physiological solution at room temperature. At the end of the stretch protocol, the smooth muscle tissues were quickly homogenized and the total protein was extracted in a lysis buffer containing a mixture of phosphatase inhibitors (NaF 10 mM; NaVO_3_ 1 mM). The protein content was quantified using BCA reagent and10 µg total protein from slack and stretched smooth muscle tissues was processed for SDS-PAGE. The different lanes were equally transferred to a PVDF membrane, and then the membrane was processed for immunobloting using specific primary antibody against Akt (polyclonal, Cell signaling), phosphorylated Akt (Ser 473, monoclonal, Cell Signaling), eNOS (polyclonal, BD Biosciences) and phosphorylated eNOS (Ser 1177, polyclonal, Cell Signaling), respectively. At the end of the western-blot protocol, the specific signal was probed using a Horseradish peroxidase substrate (Millipore). The specific signals were quantified using densitometry with Image J software and the phosphorylation levels of Akt and eNOS were corrected for the total amount of respective protein from the same extracts.

### Data Analysis

Image processing and data analysis were done with custom software written in MATLAB. Ca^2+^ sparks were counted manually and with a spark-counting software algorithm to verify the result objectively. The results are expressed as means±SEM. Contractile amplitudes are expressed relative to control (stretched to 0.2 g and absence of pharmacological intervention) values. Significant differences were determined by the Student's *t* test. Data from three groups were compared by one-way, repeated measures ANOVA and significant differences between groups determined by the Student-Newman-Keuls (SNK) test for paired comparisons.

## Results

### Ca2+ release is related to the length and time of stretch

To investigate the underlying mechanism of stretch-induced Ca2+ release in smooth muscle, we examined the properties of Ca2+ sparks at slack and stretched cell length. Stretch of single mouse bladder smooth muscle cells by 9% to 18% of the slack length triggered Ca2+ sparks and/or waves as previously reported [3]. The probability (percent of experiments or cells with Ca2+ sparks) and frequency of Ca2+ release events induced by stretch were highly related to both the degree of stretch and the time that stretch was maintained. Figure 1A shows that how single cells were stretched. Figure 1B shows linescan images from different stretch lengths; at 9% increased length, the probability of Ca2+ release events was 19.2% (5 out of 26 cells with Ca2+ release events), whereas stretch to 18% from slack produced Ca2+ release events in 82.2±7.4% (23 out of 28 cells) of cells, or a 3.1 fold increase in release probability. A similar finding was also obtained from x-y images recorded by confocal microscopy (Olympus, FV 1000, Fig. 1C). Ca2+ spark frequency was also dependent on the extent of stretch. Elongation of myocytes by 9% of resting length (ΔL = 9%) resulted in a Ca2+ spark frequency of 3.7±0.92 per second (Fig. 1B&E), while the frequency was 8.1±1.43 per second at ΔL = 18%. To examine the effect of the time of stretch on Ca2+ release events, cells were stretched by 9% (ΔL = 9%) from slack and maintained at the length for 5 min (Figure 1 D&E). Both Ca2+ spark probability and spark frequency were markedly and significantly increased after 5 min (17.2% (5 out of 29 cells with Ca2+ release) vs 79.0% (26 out of 33 cells with Ca2+ release), and 3.5±0.46 vs 8.1±1.76/second, respectively. Thus Ca2+ spark probability and frequency are a function of the length of individual myocytes, and augmented Ca2+ release is maintained after acute stretch.

### NO involvement in stretch- induced Ca2+ release in smooth muscle

**Figure 2:**
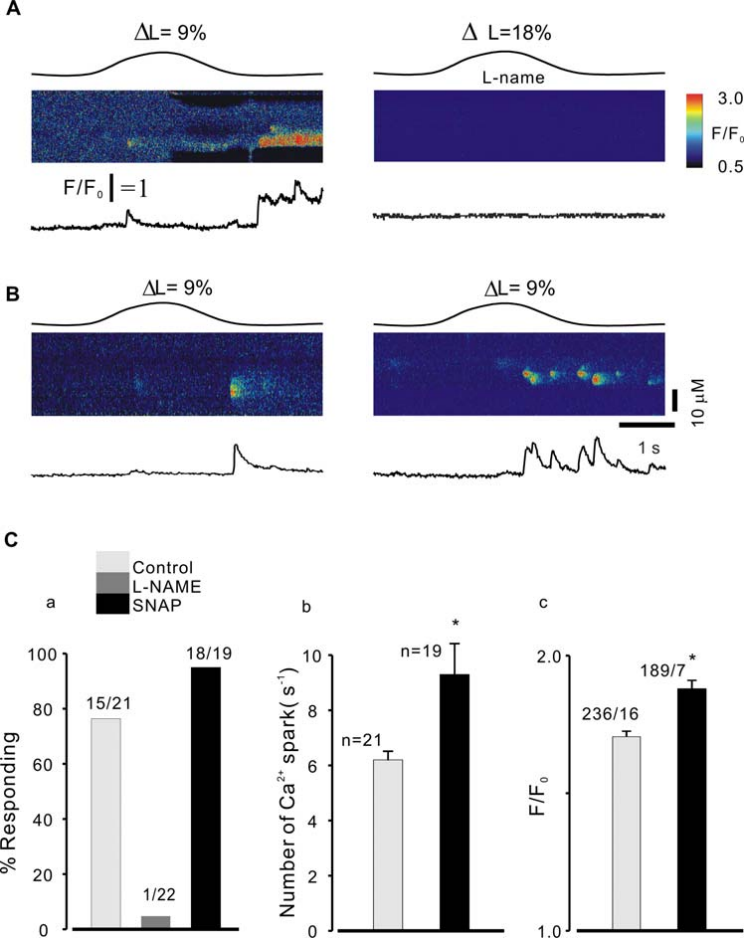
Effect of NO inhibitor and donor on SICR in smooth myocytes . A, linescan images obtained from a cell without incubation with NOS inhibitor, L-name (left), and a cell in the presence of L-name (right). Control experiment demonstrating stretch-activated Ca2+ release(left, ΔL = 9%); in the presence of L-name the Ca2+ release was completely abolished even at ΔL = 18% stretch length of cells (right). B, sample of linescan images obtained from control (without SNAP) cells and the cells exposured to SNAP, respectively. In the presence of SNAP (100 µM, right) Ca2+ spark property markedly altered by stretch the cell length to ΔL = 9% compared to the control cell. C, summary data of Ca2+ spark probability, frequency, and peak calcium. Stretch-induced Ca2+ sparks were entirely abrogated by L-NAME; in contrast, in the presence of SNAP the probability of experimental cells with Ca2+ sparks was higher than that observed in control group (without SNAP) in stretched myocytes (Ca); Ca2+ spark frequency in SNAP group was greater than that in control experiments (Cb); in addition, in the presence of SNAP the peak Ca2+ was also significantly different from that of control (Cc). The numbers in Ca and Cb indicate the number of experiments, and the numbers in Cc are sparks/experiments. * indicates P value less 0.05.

As NO synthesis has previously been implicated in stretch –induced Ca2+ release in cardiac muscle [16], we examined the effects of NOS inhibition and exogenous NO on stretch -induced Ca2+ release. As shown in Figure 2, Ca^2+^ release occurred in most of cells tested when cells were stretched by 18% (77.8%, 14 out of 18 of cells with Ca2+ release in control experiments, Fig. 2A, left), demonstrating stretch-activated Ca2+ release following cell elongation (ΔL = 9%); whereas in the presence of L-name (1 mM ), the Ca2+ release was almost completely abolished at a stretch of 18% (4.6%, 1 out of 22 cells with Ca2+ sparks; Fig. 2A, right). Moreover, exposure of cells to the NO-generating compound, *S*-nitroso-*N*-acetylpenicillamine (SNAP, 10 µM,) increased the probability of stretch -induced Ca2+ sparks further (95.0% (18 out of 19 cells tested with SNAP, Fig. 2B&C) vs 71.4% (15 out of 21 cells without SNAP). Ca2+ spark frequency increased by 50±3.6% in the presence of SNAP compared to that of control (Fig. 2B&Cb). The Fluo-4 fluorescence ratio (F/F0) was also significantly higher in the presence of SNAP than that observed in control experiments (Fig. 2Cc, 1.7±0.02 vs 1.88±0.03, P<0.05; n = spark numbers/experiments).

We further examined the effect of SNAP on CICR in voltage clamped myocytes (data not shown). When mouse bladder single cells were depolarized to −30 mV from the holding potential of −70 mV, Ca2+ sparks (Ca2+ induced Ca2+ release, CICR) occurred [21], [22]. The frequency of depolarization -induced Ca2+ sparks was markedly increased (1.5 fold, ) in the presence of SNAP, whereas neither the probability of observing sparks (94.0% (15 out of 16 experiments) versus 94.46%(17 out of 18 cells)), nor the peak change in fluorescence (1.74±0.04 versus 1.66±0.06) were significantly different. These results augment our previous findings that SICR and CICR occur through equivalent mechanisms, involving ryanodine receptors [22], indicating that NO has similar effects on both processes.

### SICR in eNOS null myocytes

**Figure 3:**
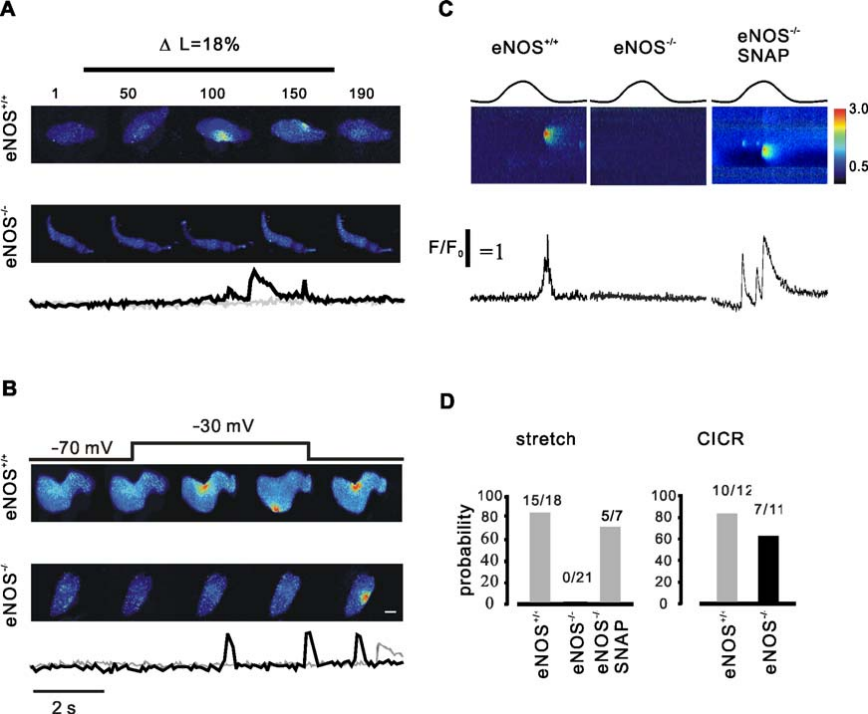
eNOS gene deletion alters the property of stretch- induced Ca2+ sparks in smooth myocytes. A, representative x-y images recorded from a wildtype (upper) and an eNOS deficient smooth myocyte (lower). The stretch-dependence of Ca2+-spark frequency is present in fluo-4-loaded smooth myocytes from the wild type (WT, 15 out of 18 experiments) but absent in those from eNOS-deficient mice (0 out of 21 experiments); the below of the images are profiles of Ca2+ ratio from WT (black) and eNOS null (gray) cells. B, sample of x-y images recorded from membrane voltage depolarized WT (upper) and eNOS knockout (lower) smooth myocytes; compared to WT cells, the eNOS deletion cells have a low Ca2+ spark probability (see also D, right). C, linescan images recorded from WT (left), eNOS null cells (middle) as well as in the presence of SNAP (right). D, summary data of Ca2+ spark probability in WT and eNOS deficient smooth myocytes.

To further examine NO involvement in the stretch-induced Ca2+ release we next examined SICR in eNOS-deficient and strain –matched control (wildtype) mice. Myocytes were loaded with fluo4 and exposed to controlled stretch as described above. As shown in Figure 3, SICR was completely eliminated in eNOS null mice. Stretch to 18% of slack length evoked SICR in the form of Ca2+ sparks in 83.3% (15 out of 18 cells) from control myocytes, whereas Ca2+ release events were never observed in x-y (Fig. 3A) or linescan (Fig. 3C) experiments in eNOS-/- myocytes exposed to the same protocol (0 out of 21 expeiments), indicating that NO formation is necessary to evoke stretch-induced Ca2+ release. We also noted that incubated the eNOS-/- with SNAP Ca2+ sparks occurred again after stretch and maintained in 71.4% of cells tested (Fig. 3C&D). Parallel CICR experiments demonstrated Ca2+ release in wildtype and eNOS deficient cells depolarized from −70 mV to −30 mV (Fig. 3B), however, the probability of evoking Ca2+ sparks by depolarization was not significant statistically though it was lower in eNOS knockout myocytes (63.6%, 7 out of11 cells)versus 83.3% in wildtype (10 out of 12 experiments, Fig. 3D, right). Analysis indicated that the kinetics of Ca2+ sparks induced by voltage depolarization was not altered between weldtype and knockout cells. Taken together, these results indicate that Ca2+ sparks induced by stretch is mediated by NO production in smooth muscle.

### Stretch regulates NO synthesis

**Figure 4:**
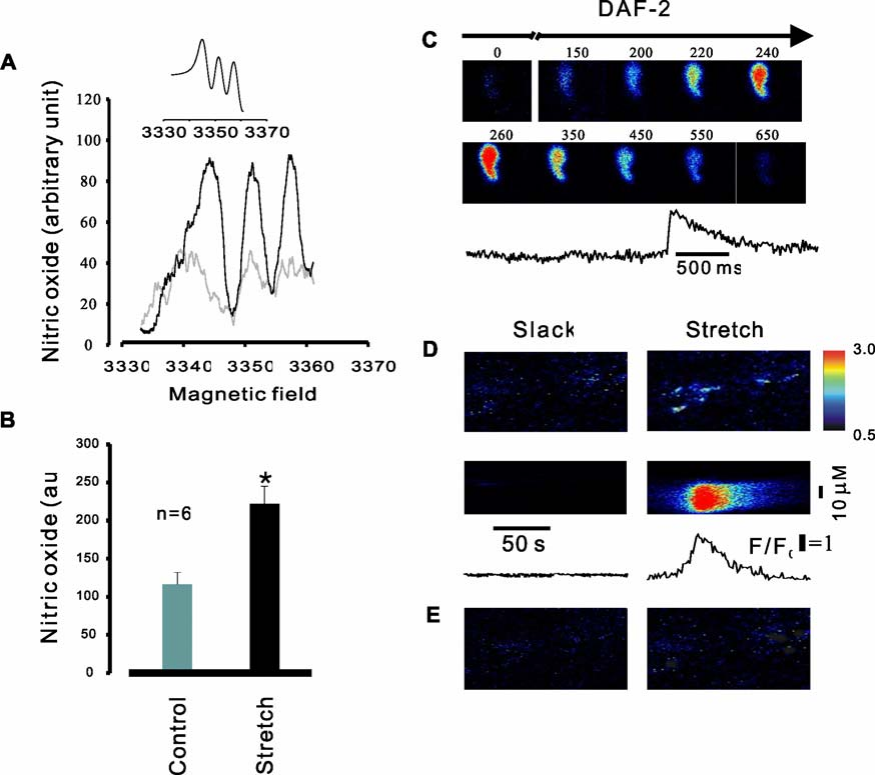
Stretch-induced NO production in single cell and intact smooth muscle tissues. A, ESR spectra of NO trapped by DETC-iron (II) complex in mouse bladder smooth muscle strips. Compared to control (slack tissue strips, gray line), NO was greatly increased in stretched tissue strips (black line). The inset shows the magnetic field range of NO in the presence of SNP. B, summary data of NO production. Note the significantly difference of NO production between the control group and stretch group; * P<0.01, n = 6. C, DAF-2 single cell experiments: by stretch the cell length to ΔL = 9% DAF-2 fluorescence transient occurred immediately. D, upper is x-y images taken from a tissue strip incubated with DAF-2; the lower is pseudo linescan images taken from a series of x-y image obtained from before (left) and after (right) stretch of the intact mouse bladder smooth muscle strip incubated with DAF-2, and at below of pseudo linescan images show NO transient profiles taken from slack and stretch. E, before ( left ) and after ( right ) stretch of tissue segment in the presence of L-name. Note that the stretch-induced NO production was completely inhibited by L-name.

To directly determine the relationship between cell stretch and NO concentration, we used electron spin resonance (ESR) spin-trapping –based detection [23], [24] in urinary bladder tissue segments exposed to rapid stretch. As shown in Figure 4 (A&B), elongation of tissue segments to about 30% of the initial length caused a 2 fold increase of NO production trapped by the diethyldithiocardamate (DETC)-iron (II) complex (221.3±24.6 vs 114.5±17.8 au). These results were confirmed and extended to single myocytes by loading with the NO-sensitive indicator 4, 5-diaminofluorescein (DAF-2). Stretching single myocytes by an average of 9% of the initial length resulted in a rapid rise in fluorescence in 5 out of 6 experiments (Fig. 4C), indicating an increased formation of NO within milliseconds after acute cell elongation. Similarly, rapid elongation of tissue segments by 20∼30% of the initial length resulted in multiple cells producing discrete DAF-2 fluorescence transients (Fig. 4D, upper). Fluorescence was not altered by stretch of unloaded cells or tissues segments or in L-NAME loaded tissue strips (Fig. 4E). Thus, eNOS activity and the cellular concentration of NO are critically regulated by mechanical forces imposed on smooth muscle.

### NO mediates stretch-induced contraction of smooth muscle

**Figure 5:**
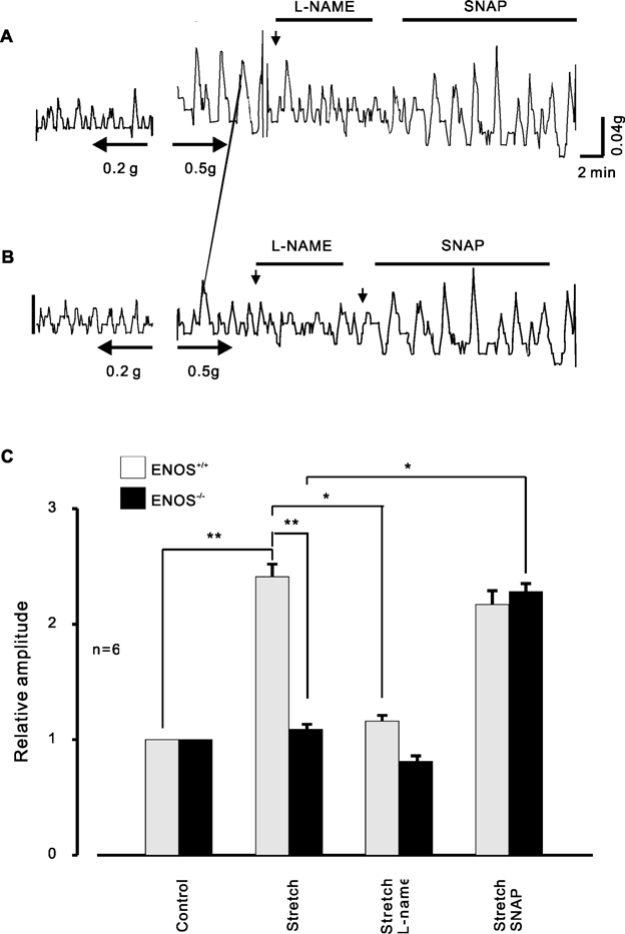
NO mediated contraction of mouse bladder smooth muscle strips. A&B, samples of spontaneous contractions recorded in wildtype (A) and eNOS deleted (B) mouse urinary bladder smooth muscle strips. After stretch to 0.5 g from the control (0.2 g), the amplitude of contraction was prominent increased in wildtype myocytes (n = 6, **p<0.01) but not in eNOS knockout myocytes; the stretch induced increase in amplitude of contraction could be inhibited by application of L-NAME (*P<0.05). In contrast to wildtype strip, eNOS deficient strip did not response to this compound but did response to SNAP (B, n = 6, *P<0.01). C, summary data of contraction amplitude. n = 6, *P<0.05, **P<0.01, respectively.

To determine the relationship between NO formation and contractile activity, the force production of detrusor tissue segments from wild type and eNOS deficient bladders was measured. Figure 5A shows a sample of experiments from wild type segments. The amplitude of contraction of eNOS+/+ bladder segments was increased 2.5 fold compared to control (p<0.01, n = 6), whereas the frequency of spontaneous contractions was significantly decreased (1.35 fold, p<0.05) in stretched tissue strips. Moreover, after exposure to L-NAME (1 mM) stretch -induced increases in contraction amplitude were abrogated in a reversible manner. In contrast to wild type tissues, neither stretch nor application of L-NAME affected the amplitude or frequency of contraction in eNOS knockout tissue strips (Fig. 5B). Finally, the NO donor SNAP increased the amplitude of contractions 2.8 fold (n = 6, P<0.01), and decreased the frequency of contractions 1.5 fold in both weldtype and knockout myocytes (Fig. 5C, P<0.05). Taken together, these experiments suggest that NO production mediates stretch- induced Ca2+ release and muscle contraction.

### Guanylyl Cyclase and PI(3) Kinase in stretch-induced Ca2+ release

**Figure 6:**
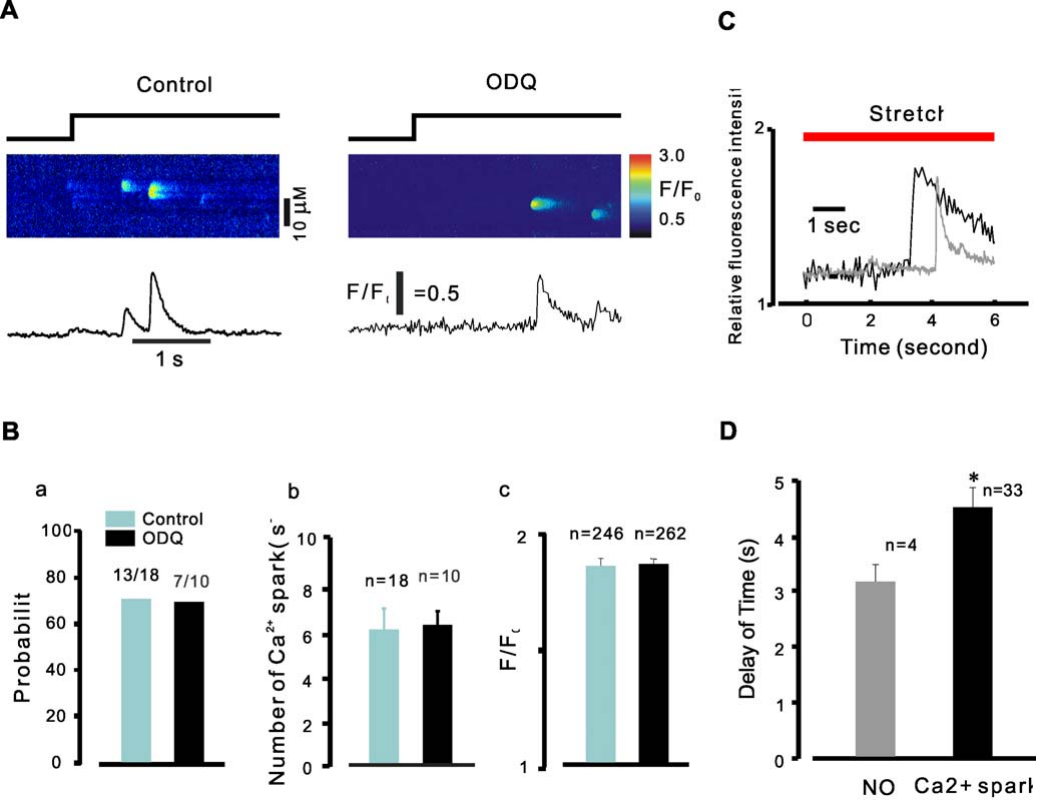
Ca2+ spark property was not altered by ODQ. A, sample of linescan images obtained from stretched smooth myocytes. Compared to control (A, left), there was no significant change of the Ca2+ spark probability (A, right), frequency, and peak Ca2+ in the presence of ODQ. B, summary data of probability (a, n = number of experiments), frequency (b, n = number of experiments), and peak Ca2+ (c, n = number of sparks) of Ca2+ sparks. C & D, profiles and summary data show the relationship of NO and stretch-induced Ca2+ spark, indicating NO always occurred prior to Ca2+ sparks (n = number of experiments, *P<0.05).

We next examined whether stretch -induced formation of NO acts through the stimulation of guanylyl cyclase (GC) and cGMP/protein kinase G (PKG). Single bladder myocytes were co-incubated with Fluo-4 AM and the GC inhibitor 1-H-[1], [2], [4,]-oxadiazolo [4,3-α]quinoxalin-1-one (ODQ, 10 µM), and stretch –induced Ca^2+^ release examined. As shown in Figure 6, stretch (ΔL = 18%) –induced Ca^2+^ sparks were not altered in myocytes exposed to ODQ 10 µM. Moreover, neither the PKG inhibitor KT5823, nor the cell-permeable cGMP analogue 8-Br-cGMP, affected Ca2+ sparks (data not shown), suggesting that NO formation did not promote Ca2+ release by activation of the classical GC/PKG pathway.

**Figure 7:**
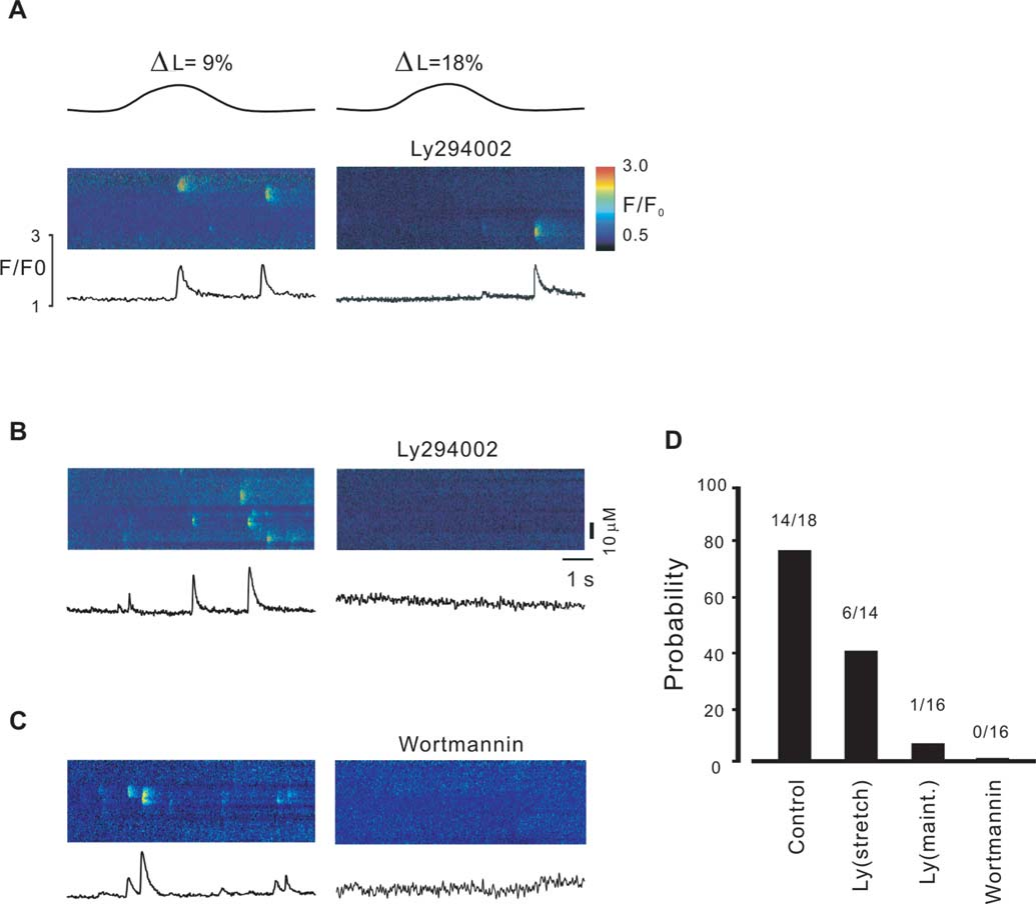
PI(3)k-Akt mediates stretch- induced Ca2+ release in smooth myocytes. Linescan images show the effect of LY492002 on Ca2+ spark at stretch length ΔL = 18%. LY492002 could not completely abrogated the immediately stretch-induced Ca2+ sparks (A), but completely abolished Ca2+ sparks occurred in stretch-maintaining stage (B). C, representatives of experiments in the absence and the presence of another PI3 kinase inhibitor, wortmannin. Like LY492002, wortmannin entirely abrogated Ca2+ sparks occurred in the stretch-maintaining stage. C, summary data of Ca2+ spark probability after sequential stretch. The numbers indicate the response experiments to stretch out of all experiments.

The stretch dependence of Ca^2+^-spark frequency has been linked to a PI(3)K-dependent signaling pathway in cardiac myocytes [16]. To determine whether a similar signaling pathway is involved in smooth muscle, PI(3)K inhibitor, LY294002 and wortaminn were used in next series of experiments. Ca2+ sparks induced by stretch were markedly reduced following incubation with LY294002 (10–30 µM). 42.9±13.7% of cells exposed to 18% stretch exhibited Ca^2+^ sparks (6 out of 14 experiments, Fig. 7A and D), compared to 6.3±6.3% of cells exposed to LY294002 30 µM(1 out of 16 experiments)in stretch maintaining stage (Fig. 7B, left and D), supporting a role of PI(3)K-dependent signaling in the signaling pathway leading to Ca^2+^ sparks. In order to confirm the pathway another PI(3)K inhibitor, wortmannin, was examined further. As shown in Fig. 7C and DE, wortmannin entirely inhibited the occurrence of stretch-induced Ca2+ sparks, confirming the PI(3)K-dependent signaling pathway of stretch induced Ca2+ release in smooth muscle.

**Figure 8:**
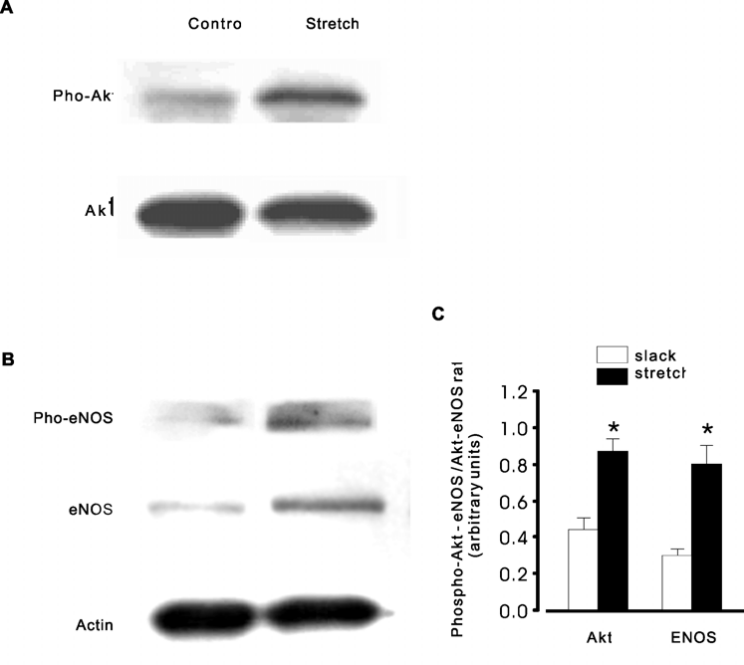
Stretch-induced Akt/eNOS phosphorylation and eNOS up-regulation in smooth muscle. Mouse bladder tissue strips were stretched to and maintained at 30% of slack length at least for 10 min, and then frozen. A & B, representative western blots showing Akt and eNOS phosphorylation and expression. Stretching of smooth muscle strips induced a significant increase in the phosphorylation of both Akt (204±28.6%) and eNOS (258±36.8%), respectively. It was noted that the eNOS protein level was also up-regulated by stretch. C, densitometry for ratio of Akt and eNOS phosphorylation to Akt and eNOS expression. Results are expressed as mean±SEM (n>8 mice for each group). *P<0.05 compared with control (slack).

To further prove the NO dependence of stretch-induced Ca2+ release via activation of PI(3)K/Akt axes in mooth muscle, we detected the phosphoryaltion of Akt and eNOS levels. As shown in figure 8 both phospho-Akt (A) and phospho-eNOS (B) levels were significantly changed by stretch of smooth muscle (increased 204±28.6% for Akt and 258±36.8% for eNOS, respectively). The stretch –induced phosphorylation of both Akt and eNOS was complicated abrogated by wortaminn (data not shown). Finally, it was notable that the eNOS protein level was also up-regulated by 160% by stretch in smooth muscle (B).

## Discussion

Spatially and temporally restricted SR Ca2+ release events in the form of Ca2+ sparks play a crucial role in the regulation of sarcolemmal, Ca2+ activated ion channels [25]. We and others have shown that elongation of cells increases the probability of Ca2+ release events in myocytes [3], [16]. Here we have examined the mechanism underlying stretch-induced Ca2+ release in smooth muscle. Our main finding is that stretch-induced Ca2+ sparks are both the stretch length and time dependent (Fig. 1) and are mediated by the generation of NO and that the action of NO is independent of the NO-sGC-cGMP pathway. The augmentation of Ca2+ spark probability and frequency observed after cell elongation were abolished by incubation with the NO inhibitor, L-NAME, and enhanced by the NO donor, SNAP (Fig. 2, 3). Moreover, elongation of smooth muscle resulted in an increase in NO production in tissue segments and single cells (Fig. 5) and eNOS gene inactivation resulted in the loss of SICR, but not CICR (Fig. 3). We have also demonstrated a functional role for NO coupling to SICR: stretch –induced contractions were inhibited by L-NAME and enhanced by SNAP (Fig. 5). The production of NO preceded the induction of Ca2+ sparks (Fig. 6 C&D), but its action was not dependent on soluble guanylyl cyclase activity and the activation of PKG, as SICR occurred in the presence of sGC and PKG inhibitors, and was not augmented by a cell permeant cGMP analogue (Fig. 6). On the other hand, release was inhibited by the PI3 kinase inhibitor LY492004 and wortmannin (Fig. 7). Moreover, phosphorylation assay of Akt and eNOS demonstrated that both phospho-Akt and phospho-eNOS levels were greatly up-regulated by stretch (Fig. 8), suggesting the activation of PI(3)K/Akt pathway by stretch further.

These findings establish NO as a critical coupling molecule linking membrane tension to SR Ca^2+^ release. NO has been shown to increase the activity of ryanodine receptors in cardiac [14], [16] and skeletal [18], [26] muscle. As the stimulation of guanylyl cyclase by NO is a prominent smooth muscle relaxant mechanism [27], the role of NO in SICR seems counter intuitive. However, similar to cardiac muscle, SICR is independent of the activation of guanylyl cyclase and PKG, indicating a compartmentalization of NO signaling. Wees et al linked stretch –induced NO formation and Ca2+ release to the stimulation of integrins [14], presenting evidence that release occurs through the stimulation of cardiac type, RYR2, ryanodine receptors. We have previously shown that SICR in smooth muscle also occurs through the activation of RYR2 SR channels [3], [22]. it has been known that FKBP12.6 is closely associated with RYR2 [28], we, therefore, further examined SICR in FKBP12.6 deletion urinary bladder smooth myocytes in order to supporting SICR occurs through activation of RYR2 in smooth muscle. The results indicated that Ca2+ spark property and kinetics were significantly altered by stretch in FKBP12.6 null myocytes compared to that in wildtype myoccytes (data not shown). Thus SICR in smooth muscle bears essential similarities to that of cardiac muscle. Although recent work in mouse embryo bladder myocytes demonstrated that RYR1 is required for depolarization-induced Ca2+ spark [29], our conclusion that stretch-induced Ca2+ spark was mediated by activation of RYR2 does not oppose the voltage depolarizing induced Ca2+ release through RYR1 in mouse embryo bladder smooth muscle. This was also proved by the parallel experiments of stretch and voltage depolarization carried out in eNOS-/- myocytes (Fig. 3) in the present study that stretch did not cause Ca2+ spark but membrane voltage depolarization did, suggesting that the underlying mechanisms of stretch-induced Ca2+ spark and voltage depolarization induced Ca2+ spark are different.

It remains unclear how NO affects Ca^2+^ release via RyR2. The activation of ryanodine receptors through *S*-nitrosylation has been demonstrated extensively *in vitro* [30], [31], although definitive evidence for physiological ryanodine receptor regulation is limited. In this regard, future studies on mechanical activation of muscle should focus on the mechanism by which the action of NO remains tightly linked to RYR2 activation.
